# Divergent immune ecosystems in HPV-associated and HPV-independent lower anogenital tract malignancies

**DOI:** 10.3389/fimmu.2026.1937031

**Published:** 2026-09-22

**Authors:** Hongmei Wang, Zhifeng Sun, Yanlin Zhang, Hongli Zhu

**Affiliations:** 1 School of Basic Medicine, Shaanxi University of Chinese Medicine, Xianyang, Shaanxi, China; 2 Department of Gynecology, Affiliated Hospital of Shaanxi University of Chinese Medicine, Xianyang, Shaanxi, China; 3 Hubei Clinical Research Center for Reproductive Medicine, Shiyan, Hubei, China; 4 Hubei Key Laboratory of Wudang Local Chinese Medicine Research, Shiyan Key Laboratory of Reproduction and Genetics (Renmin Hospital), Hubei University of Medicine, Shiyan, Hubei, China; 5 Innovation Team for Prevention and Treatment of Neurodegenerative Diseases with Traditional Chinese Medicine, Shaanxi University of Chinese Medicine, Key Laboratory for Prevention and Treatment of Cerebrospinal Diseases, Administration of Traditional Chinese Medicine of Shaanxi Province, Xianyang, Shaanxi, China

**Keywords:** antigen presentation, HPV-independent carcinogenesis, human papillomavirus, immune checkpoint inhibitor, lower anogenital tract malignancies, precision immunotherapy, spatial transcriptomics, tumor immune microenvironment

## Abstract

Lower anogenital tract malignancies comprise anatomically adjacent but biologically heterogeneous epithelial cancers of the cervix, vulva, vagina, anal canal and penis. This narrative and conceptual Review used a transparent literature search to examine how human papillomavirus (HPV) etiology intersects with tumor lineage, somatic alterations, antigen-presentation competence, immune-cell state and spatial organization. Evidence quality is heterogeneous: cervical cancer is supported by the largest genomic, single-cell, spatial and randomized-trial literature, whereas vaginal, vulvar and penile cancers remain constrained by small retrospective cohorts and cross-site extrapolation. HPV-associated tumors retain non-self E6/E7 antigens, but viral transcription does not ensure peptide presentation, dendritic-cell priming or epithelial access by functional T cells. HPV-independent tumors lack constitutive viral antigens yet may generate mutation- or differentiation-derived immunity and checkpoint-restrained myeloid–lymphoid niches. We therefore distinguish etiological status from functional immune phenotype and critically appraise three spatial architectures—an inflamed/epithelial-penetrating pattern, a stromally excluded pattern, and an immune-desert pattern—together with an immunosuppressive functional overlay that may occur in any architecture. Clinical evidence is graded separately from mechanistic inference: immune checkpoint blockade has established roles in defined cervical and anal cancer settings, whereas therapeutic vaccines, tumor-infiltrating lymphocytes, T-cell receptor-engineered therapies, myeloid or stromal sensitization and artificial-intelligence-guided selection remain investigational or context dependent. A three-tier biomarker framework is proposed, separating routine clinical assays from extended translational profiling and exploratory single-cell, spatial and artificial-intelligence methods. This framework is hypothesis-generating, not a validated treatment-selection algorithm; assay combinations, thresholds and incremental clinical utility require prospective, multicenter and site-stratified validation.

## 1 Introduction

Lower anogenital tract malignancies include invasive epithelial cancers of the uterine cervix, vulva, vagina, anal canal and penis. Their anatomical proximity and shared susceptibility to oncogenic human papillomavirus (HPV) have encouraged their treatment as a related disease family. That grouping is useful for prevention and for understanding viral oncogenesis, but it can become misleading when it overrides epithelial lineage, precursor lesion, site-specific stroma, lymphatic anatomy and established treatment standards. Cervical squamous cell carcinoma, usual-type endocervical adenocarcinoma, gastric-type endocervical adenocarcinoma and keratinizing vulvar or penile squamous carcinoma are not biologically interchangeable even when discussed under the same anogenital label (1–4).

Persistent infection with a carcinogenic HPV genotype drives almost all conventional cervical cancers and most anal squamous cell carcinomas, but contributes more variably to vaginal, vulvar and penile squamous carcinomas. HPV16 predominates at most non-cervical sites, whereas cervical tumors display a broader genotype distribution. The resulting burden is shaped by vaccination, screening, sex, age, immune status, geography and access to care. People living with HIV and solid-organ transplant recipients have particularly elevated incidences of several HPV-related cancers, illustrating the importance of durable immune surveillance (5, 6).

Contemporary pathology increasingly separates HPV-associated from HPV-independent disease. This distinction is most mature in vulvar squamous cell carcinoma and endocervical adenocarcinoma, where HPV/p16 and p53-informed classifications identify clinically distinct entities (7–9). However, the terms HPV-positive, p16-positive, transcriptionally active HPV and HPV-associated are not synonymous. HPV DNA may be incidental or transcriptionally silent; block-type p16 is an etiological surrogate whose specificity varies by site and histology; and a negative assay can reflect technical failure rather than genuine HPV independence. Etiological assignment therefore requires a site-aware integration of morphology and molecular testing.

The immunological consequences of HPV are paradoxical. E6 and E7 are non-self proteins required for maintenance of transformation and are therefore attractive antigens. Yet established cancers have already passed through antiviral and antitumor immune selection. Viral antigenicity may coexist with defective innate sensing, impaired dendritic-cell maturation, HLA class I loss, regulatory lymphocytes, suppressive macrophages, metabolic stress and fibroblast-rich exclusion (10–13). Conversely, HPV-independent tumors may generate clonal neoantigens or altered differentiation antigens and can contain active, although frequently restrained, immune responses. The shorthand that HPV-associated tumors are uniformly immune-hot and HPV-independent tumors uniformly immune-cold is therefore mechanistically inadequate.

This Review has three objectives. First, it defines which histological entities can be compared within a lower anogenital framework and where lineage- or site-specific interpretation must take precedence. Second, it evaluates molecular, single-cell, spatial and clinical evidence according to what each method can actually establish. Third, it proposes a tiered, explicitly hypothesis-generating biomarker framework that separates routine clinical assays from translational and exploratory measurements. Because most direct evidence derives from cervical cancer, that disease is treated as the reference model; conclusions for rarer sites are not inferred automatically from cervical data.

## 2 Review methodology

This article is a narrative and conceptual Review rather than a systematic review or meta-analysis. We searched PubMed/MEDLINE, Embase, Web of Science Core Collection and Scopus from database inception through 25 July 2026. ClinicalTrials.gov, the WHO International Clinical Trials Registry Platform, the US Food and Drug Administration and European Medicines Agency websites were consulted for trial status and jurisdiction-specific regulatory information. Principal concepts combined terms for HPV, p16, E6/E7, HPV-independent disease, cervix, vulva, vagina, anus, penis, squamous cell carcinoma, adenocarcinoma, tumor immune microenvironment, antigen presentation, HLA, B2M, dendritic cells, macrophages, tertiary lymphoid structures, single-cell RNA sequencing, single-nucleus RNA sequencing, spatial transcriptomics, immunometabolism, immune checkpoint inhibition, therapeutic vaccination, adoptive cell therapy, circulating tumor HPV DNA, digital pathology and artificial intelligence.

English-language original studies, randomized trials, prospective cohorts, well-annotated retrospective cohorts, pathological classification studies, systematic reviews and authoritative tumor classifications were eligible. Conference abstracts were used only when no full report was available and were explicitly labeled; preprints were not used as core evidence. Regulatory documents were cited only to describe a named jurisdiction and date, not as universal clinical standards. Case reports were excluded unless they supplied unique mechanistic evidence in an exceptionally rare setting.

Landmark studies were defined as those establishing an etiological classification, practice-changing randomized result or first direct demonstration of a mechanism. Representative studies were selected to illustrate major anatomical sites, technologies and geographical settings. Confirmatory studies were included when they tested the same biological proposition in an independent cohort or by an orthogonal method. When cohorts overlapped, the largest or most mature report was used for the principal clinical endpoint; related publications were retained only when they supplied distinct molecular or longitudinal analyses. Evidence from other solid tumors was used selectively for general mechanisms such as TGF-β-associated exclusion, extracellular-matrix restriction or metabolic competition and is identified as extrapolative rather than direct lower anogenital evidence.

No formal pooled effect estimate or risk-of-bias score was calculated. Instead, claims were graded narratively as: (i) regulatory or guideline-linked indication within a named jurisdiction; (ii) randomized clinical evidence; (iii) prospective disease-specific activity; (iv) prognostic association; (v) computational or spatial association; (vi) functional perturbation; or (vii) unvalidated mechanistic hypothesis. This hierarchy was used to prevent computational ligand–receptor inference or HPV prevalence within a trial population from being interpreted as functional causality or predictive biomarker evidence.

The authors independently reviewed the search decisions, evidence appraisal, factual statements, citations, references, clinical data, scientific interpretation, final wording, and visual elements and take full responsibility for the final content.

## 3 Scope and site-specific etiological classification

The comparative framework includes invasive cervical squamous cell carcinoma and usual-type HPV-associated endocervical adenocarcinoma; HPV-independent cervical adenocarcinomas, particularly gastric-type, clear-cell and mesonephric carcinomas; and invasive squamous cell carcinomas of the vulva, vagina, anal canal and penis. Precursor lesions are discussed only when they clarify the route to invasion. Non-epithelial malignancies, neuroendocrine carcinomas, melanoma and sarcoma are outside the main scope. Cross-site comparison is biologically justified when tumors share transcriptionally active high-risk HPV and E6/E7 dependence or when a common immune bottleneck, such as HLA loss, is directly demonstrated. Site- or lineage-specific interpretation takes precedence for cervical adenocarcinoma, chronic-dermatosis-associated vulvar cancer and other tumors whose developmental origin and genomic drivers differ substantially.

Etiological classification should begin with morphology and an adequate tumor sample. HPV DNA testing detects viral genomic material but does not prove transcriptional activity. PCR-based assays offer genotype sensitivity but may detect incidental infection and can be affected by DNA quality or limited genotype coverage. DNA *in situ* hybridization adds localization but is less sensitive at low copy number. E6/E7 RNA *in situ* hybridization provides cellular localization of transforming transcription and has been analytically compared with PCR, DNA *in situ* hybridization and p16 immunohistochemistry, but performance still depends on fixation, RNA preservation and assay coverage (14).

Block-type p16 immunohistochemistry is a practical surrogate for transforming HPV in cervical squamous carcinoma and many vulvar or penile squamous tumors, but it is not equally specific across sites and histologies. Non-HPV mechanisms can activate p16, and some transcriptionally active HPV tumors may lack classic block staining. p53 immunohistochemistry is not an HPV assay; it is used to identify mutation-pattern abnormalities and is particularly important in HPV-independent vulvar carcinoma. A site-specific algorithm and potential discordances are summarized in **Table 1**. Central pathology review is recommended in trials that pool rare sites, because misclassification of origin or lineage can overwhelm biological comparisons.

**Table 1:** Site-specific classification of HPV-associated and HPV-independent lower anogenital tract malignancies.

Site/entity	Preferred etiological evidence	Role of p16/p53	Important discordances or false negatives	Interpretive rule
Cervical SCC/usual-type adenocarcinoma	Morphology plus validated high-risk HPV; E6/E7 RNA when discordant	Block-type p16 is usually supportive; p53 is not an HPV test	Poor RNA preservation; assay coverage; low tumor; non-cervical origin	Use HPV-associated only when morphology and virological evidence are concordant (3, 14)
Gastric-type, clear-cell or mesonephric cervical adenocarcinoma	Lineage-defining morphology; absence of etiologically active HPV	p16 often non-block in gastric type; p53 may be abnormal	Occasional non-specific p16; site misclassification	Prioritize lineage-specific diagnosis over a generic HPV-negative category (8, 9)
Vulvar SCC	Morphology plus p16 and/or HPV RNA; integrate p53	p16 block supports HPV association; p53 pattern stratifies HPV-independent disease	p16 is not perfectly specific; p53-wild-type HPV-independent group is heterogeneous	Use three-tier HPV/p53 classification with central review in trials (7, 45)
Vaginal SCC	Confirm primary vaginal origin; site-appropriate HPV DNA/RNA plus p16	p16 supports but does not alone prove etiological HPV	Prior cervical/vulvar disease; prior radiotherapy; small biopsy	Do not extrapolate cervical classification without confirming origin (50)
Anal SCC	p16 plus HPV-specific testing when etiological precision is required	p16 is a practical surrogate but discordance exists	Limited genotype coverage; tissue quality; rare HPV-independent disease	Report exact assay and avoid equating p16 with active E6/E7
Penile SCC	Histology plus p16 and HPV DNA/RNA where available	Block p16 supports association; p53 may inform nonviral pathway	Histological composition and assay definitions vary	Use HPV-associated, HPV-positive and p16-positive as distinct terms (4, 51)

Evidence categories and priority labels are defined in the main text; author-proposed research tiers are not clinical guidelines.

Throughout this Review, HPV-associated denotes a tumor supported by morphology and validated evidence of etiologically relevant HPV, preferably transcriptionally active infection or a site-appropriate p16/HPV combination. HPV-positive is reserved for the specific assay result being reported. p16-positive describes immunohistochemistry only. HPV-independent denotes an integrated biological diagnosis rather than a single negative laboratory result.

## 4 Divergent oncogenic programs and convergence on immune escape

HPV-associated and HPV-independent tumors differ most clearly in the route to malignancy and the source of candidate antigens, not in a guaranteed final immune phenotype (**Figure 1**). Persistent high-risk HPV infection maintains E6 and E7 expression, disrupts p53 and RB-family control and promotes genomic instability. Viral integration is common in invasive cervical cancer but is neither universal nor obligatory; it can disrupt viral regulation, create viral–host transcripts, alter local copy number and modify nearby enhancers (15, 16). APOBEC activity may initially reflect antiviral defense but can become a mutational process that contributes PIK3CA alterations and intratumoral heterogeneity.

**Figure 1:**
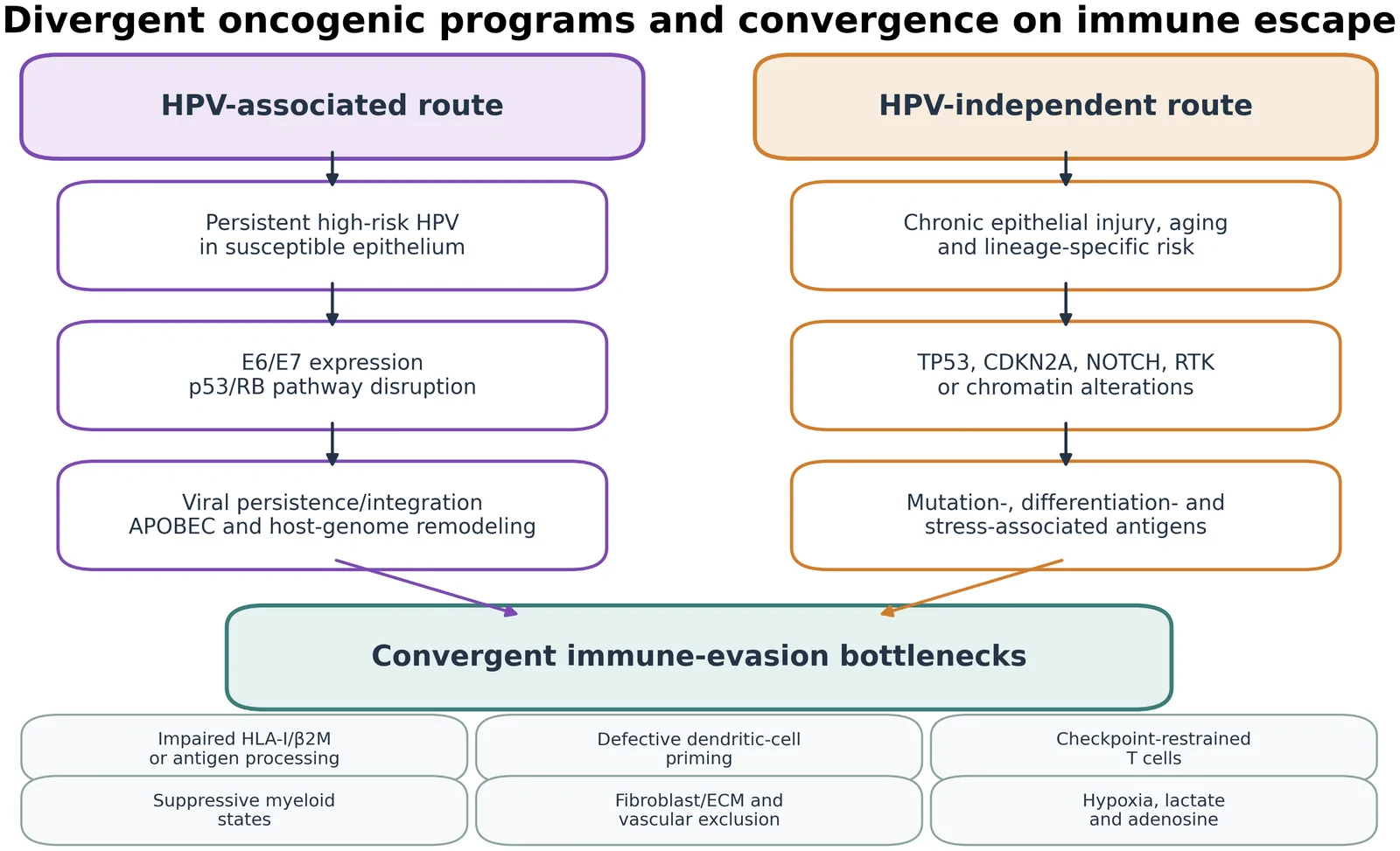
Divergent oncogenic programs and convergence on immune escape. HPV-associated tumors arise through persistent high-risk HPV infection, E6/E7 activity and host-genome remodeling. HPV-independent tumors arise through chronic epithelial injury, aging-associated clonal selection and lineage-specific genomic alterations. Both routes can converge on impaired HLA-I/β2-microglobulin or antigen processing, defective dendritic-cell priming, checkpoint-restrained T cells, suppressive myeloid states, fibroblast/extracellular-matrix and vascular exclusion, and hypoxia-, lactate- or adenosine-associated dysfunction. The diagram is qualitative and contains no quantitative estimate of HPV-attributable frequency.

HPV immune evasion operates at multiple levels. E6 and E7 interfere with pattern-recognition receptor, cGAS–STING, interferon-regulatory-factor, NF-κB and JAK–STAT signaling. Viral proteins and associated epigenetic changes reduce chemokines needed for Langerhans-cell or dendritic-cell recruitment and can suppress HLA class I antigen processing. The tumor microenvironment adds IL-10, TGF-β, prostaglandin E2, suppressive myeloid cells and regulatory T cells. Interferon-γ may induce PD-L1 and IDO1 as adaptive resistance, whereas prolonged interferon exposure can select more durable resistance programs. The resulting state is frequently antigen-rich but presentation-, priming- or trafficking-defective rather than simply immune-hot.

HPV-independent tumors arise through heterogeneous combinations of chronic epithelial injury, aging-associated clonal selection, TP53 or CDKN2A dysfunction, NOTCH-family alterations, TERT activation, receptor tyrosine kinase signaling and chromatin dysregulation. They lack constitutive E6/E7 antigens but can express clonal mutation-derived peptides, cancer–testis antigens, altered differentiation proteins, splice products and stress-associated self-antigens. A clonal host neoantigen could, as a mechanistic possibility, support stronger immunity than a poorly processed viral peptide, but this cannot be inferred from mutation count alone and requires demonstration of natural processing, HLA presentation and T-cell recognition.

Both etiological routes converge on a limited set of bottlenecks: HLA-I or β2-microglobulin loss, defective antigen processing, inadequate dendritic-cell priming, checkpoint-restrained T cells, suppressive myeloid states, fibroblast/extracellular-matrix and vascular exclusion, and hypoxia-, lactate- or adenosine-associated metabolic dysfunction. These convergent mechanisms are biologically plausible treatment targets, but only some have been validated directly in lower anogenital tumors.

## 5 Cervical cancer as the reference immune-oncology model

Cervical cancer provides the deepest evidence base because it combines a nearly universal viral etiology for conventional histologies, large genomic cohorts, accessible precursor lesions, paired treatment samples and multiple randomized immunotherapy trials. It should therefore anchor mechanistic synthesis without being allowed to dictate conclusions for every other site.

The transformation zone contains metaplastic and reserve-cell populations exposed to hormonal, inflammatory and mechanical influences. Within HPV-associated disease, squamous and glandular tumors differ in developmental origin and immune architecture. The Cancer Genome Atlas identified keratin-high and keratin-low squamous groups, adenocarcinoma-enriched clusters, recurrent PIK3CA, EP300, FBXW7, PTEN and HLA alterations, and a small HPV-negative, endometrial-like group (15). Thus, HPV dependence is an etiological axis rather than a complete molecular subtype.

Historical immunohistochemistry established that cervical tumors may lose classical HLA class I and can express non-classical HLA-E, both of which can limit cytotoxic recognition (17–19). These findings remain relevant to checkpoint blockade, therapeutic vaccination and HLA-restricted cellular therapy. Importantly, an HLA-intact result by routine staining does not exclude allele-specific loss, β2-microglobulin disruption or impaired peptide processing. Conversely, reduced HLA expression may be reversible when caused by transcriptional or interferon-dependent suppression rather than irreversible genomic deletion.

Dendritic-cell dysfunction provides a second bottleneck. Cervical pre-neoplastic and malignant tissue can generate tolerogenic dendritic-cell states, while HPV-associated signaling reduces recruitment and costimulation (20). A recent functional study identified leukemia inhibitory factor as an E6/E7-linked mediator that suppressed type I interferon from plasmacytoid dendritic cells and CXCL9 from macrophages; inhibiting the pathway restored immune recruitment and increased checkpoint sensitivity in experimental models (21). This study supports a causal route from viral oncogene expression to myeloid dysfunction, but it does not establish LIF blockade as a clinical standard.

Single-cell and spatial studies have resolved additional heterogeneity. Ou and colleagues analyzed five frozen cervical samples by single-nucleus RNA sequencing and 18 samples by high-resolution spatial profiling, reporting spatially restricted LGALS9 and IDO1 programs rather than uniform overexpression of all inhibitory genes (22). Xia and colleagues integrated single-cell and spatial data across stages of cervical carcinogenesis and reported expansion of SPP1-positive macrophages and inferred SPP1–CD44 communication during invasion (23). Su and colleagues analyzed seven untreated cervical biopsies across single-cell and spatial platforms; the single-cell component included only four fresh tumors, two HPV-positive and two HPV-negative, while spatial spots contained mixtures of many cells. Their ANXA1–FPR and MDK–LRP1 results are therefore computationally inferred communication signals, not demonstrated receptor dependence (24).

Other cervical single-cell datasets describe changing cytotoxic, proliferative, memory-like, regulatory and exhausted T-cell states across premalignant lesions, invasive tumors and metastatic lymph nodes, as well as differences between squamous carcinoma and adenocarcinoma (25–27). These studies support immune-state heterogeneity but do not permit patient-level prevalence estimates when thousands of cells are treated as independent observations. Patient, not cell, is the biological replicate. Copy-number-based identification of malignant epithelial cells and pseudotime trajectories are useful computational models, but neither proves lineage ancestry or directional differentiation.

Treatment further changes the ecosystem. Paired analyses after chemoradiotherapy indicate shifts in lymphoid, myeloid and malignant-cell programs, but the direction and magnitude depend on sampling time, radiation field, corticosteroid exposure and residual tumor content (28). Baseline and post-treatment specimens should therefore not be pooled without explicit modeling of treatment state. Longitudinal circulating tumor HPV DNA may provide a less invasive measure of residual disease, but it does not identify the cellular mechanism of resistance.

The cervical tumor microenvironment also contains metabolically distinct regions. Spatial and molecular studies implicate hypoxia, lactate-associated programs, altered lipid metabolism and adenosine signaling (29, 30). Lactate can impair T- and NK-cell function in general solid-tumor models, while glucose competition and adenosine can limit effector activity (31–33). In cervical cancer, however, most spatial metabolic associations remain observational; direct perturbation and clinical validation are limited. Metabolic markers should therefore be interpreted as candidate bottlenecks rather than established predictors.

B cells and tertiary lymphoid structures are emerging as another dimension. Cervical cohorts have associated mature tertiary lymphoid organization with favorable immune contexture or outcome, but structure definitions, tissue regions and scoring methods differ (34). General solid-tumor studies show that organized B-cell/T-cell niches can correlate with checkpoint response (35, 36), yet this association cannot be transferred automatically to cervical treatment selection. Prospective studies should distinguish mature tertiary lymphoid structures from dispersed B cells or plasma cells and should test whether they add information beyond stage, PD-L1 and established pathology.

HPV-independent cervical malignancies require lineage-specific analysis. True HPV-independent cervical squamous carcinoma is rare and vulnerable to false-negative or site-misclassification errors. Gastric-type adenocarcinoma, clear-cell carcinoma and mesonephric carcinoma are established HPV-independent entities but differ from each other. Gastric-type adenocarcinoma frequently harbors TP53, STK11, KRAS, CDKN2A and ERBB2 alterations, displays aggressive dissemination patterns and variably expresses PD-L1, B7-H3 or TIM-3 (37–39). These data justify separate molecular and immune stratification; they do not justify grouping all HPV-independent cervical adenocarcinomas into one immune class.

## 6 Site-specific immune ecosystems beyond the cervix

Direct evidence outside the cervix is thinner and more heterogeneous. Anal and vulvar cancer have meaningful etiological and immune datasets, whereas dedicated vaginal and penile single-cell or spatial cohorts remain limited. Each section therefore separates direct site-specific evidence from extrapolation.

### 6.1 Anal squamous cell carcinoma

Most anal squamous cell carcinomas are HPV16-associated, and incidence is markedly increased in people living with HIV and transplant recipients. HPV-negative or p16-negative tumors are uncommon but enriched for disruptive TP53 alterations and have shown inferior chemoradiotherapy outcomes (40–43). Poor outcome does not by itself prove an immune desert; defective apoptosis, hypoxia, rapid repopulation and stromal protection may contribute independently of baseline immune density.

A multidimensional pretreatment study identified an inflamed-resistant state in which interferon-α, interferon-γ, TNF–NF-κB and epithelial–mesenchymal transition programs coexisted with regulatory T cells and CXCL2-associated suppression (44). This finding distinguishes acute, spatially directed cytotoxic immunity from chronic inflammatory adaptation. Single-cell and spatial atlases of anal cancer remain less mature than those of cervical cancer, and future studies should report HIV control, CD4 count, prior treatment and biopsy location rather than treating immune status as a binary covariate.

### 6.2 Vulvar squamous cell carcinoma

Vulvar squamous cell carcinoma provides the clearest parallel model of HPV-associated and HPV-independent squamous carcinogenesis. A three-tier classification—HPV-associated, HPV-independent/p53-abnormal and HPV-independent/p53-wild-type—has prognostic and pathological support (7, 45, 46). The p53-abnormal group is frequently linked to lichen sclerosus or differentiated vulvar intraepithelial neoplasia and alterations involving TP53, CDKN2A, TERT, CCND1, FAT1 or NOTCH. The p53-wild-type HPV-independent group is heterogeneous and should not be treated as a biologically neutral intermediate.

Immune findings directly challenge the HPV-positive/hot versus HPV-independent/cold dichotomy. A four-case spatial transcriptomic study reported etiological differences in epithelial, collagen and inflammatory programs, but its sample size is insufficient to establish population prevalence (47). Molecular and multiplex imaging of HPV-independent vulvar carcinoma demonstrated CD8-positive/PD-1-positive T cells near CD68-positive/PD-L1-positive macrophages and identified immune-response genes associated with non-recurrence in an exploratory cohort (48). Separate work found an immune-infiltrated vulvar subset with active signaling that could be considered for neoadjuvant checkpoint studies (49). These are prognostic or spatial associations, not validated predictive classes.

Lichen sclerosus adds a chronic inflammatory and fibrotic field that may both recruit immune cells and generate TGF-β-, collagen- or macrophage-mediated exclusion. This dual effect is a reason to measure epithelial penetration and myeloid topology rather than relying on total T-cell density.

### 6.3 Vaginal carcinoma

Primary vaginal cancer is rare and most commonly squamous. A worldwide study detected HPV DNA in a substantial proportion of invasive vaginal cancers, with HPV16 dominant, but HPV DNA prevalence does not by itself define transcriptionally active disease (50). Prior cervical or vulvar HPV-associated neoplasia is common, creating diagnostic and field-effect complexity.

Dedicated genomic, single-cell and spatial studies are scarce. The vaginal wall differs from the cervix in epithelial thickness, stroma, microbiota and lymphatic drainage, while prior hysterectomy or pelvic radiotherapy may substantially remodel immune and vascular compartments. Accordingly, statements about vaginal immune architecture should be labeled as extrapolation unless derived from vaginal tissue. Central pathology review, E6/E7 RNA or site-appropriate HPV testing, p16/p53 assessment, prior-radiation history and multicenter tissue collection are prerequisites for meaningful studies.

### 6.4 Penile squamous cell carcinoma

Penile squamous carcinoma also follows HPV-associated and HPV-independent pathways. HPV-associated disease is enriched in basaloid and warty histologies and often shows block-type p16, whereas HPV-independent keratinizing tumors are linked to phimosis, lichen sclerosus, chronic balanitis and alterations in TP53, CDKN2A, NOTCH and differentiation pathways. Meta-analyses suggest that HPV or p16 status can be prognostic, but heterogeneity in assays and histological composition remains substantial (51).

Direct immune data show that viral antigenicity does not guarantee an immune-rich environment. In one cohort, HPV-positive penile tumors contained fewer infiltrating T lymphocytes, and patient-derived dendritic cells showed lower CD86 and impaired lymphocyte stimulation (52). Single-nucleus and spatial profiling of six patients reported a LAMC2-high basal/stem-like malignant population at invasive boundaries and inferred laminin–ITGA6/ITGB4 interactions; the same study associated HPV-positive basal-like states with suppression of T-cell and macrophage functions (53). These interaction results are computational and spatially associative rather than proof that inhibiting the axis will restore immunity.

Other studies have related immune infiltration or plasma cells to outcome (54, 55), while retrospective treatment cohorts suggest that HPV status may enrich for response to PD-1-based combinations (56). These signals require prospective validation and should not be interpreted as proof that HPV status is predictive. HLA class I expression also varies in penile cancer and may be more directly relevant to HPV-targeted cellular therapy than HPV DNA alone (57).

Site- and etiology-specific molecular and immune characteristics are summarized in **Table 2**.

**Table 2:** Site- and etiology-specific molecular and immune characteristics.

Site/group	Dominant context	Representative tumor program	Candidate antigen source	Immune patterns (not universal)	Interpretive boundary
Cervix, HPV-associated	Transformation-zone high-risk HPV	E6/E7; APOBEC; PI3K; epigenetic and HLA alterations	E6/E7 plus host neoantigens	Interferon/checkpoint programs, dysfunctional DCs, SPP1+ macrophages, variable T-cell penetration	Cervical evidence is deepest but still heterogeneous (15, 22)
Cervix, HPV-independent	Gastric-type, clear-cell, mesonephric or rare true nonviral SCC	TP53, STK11, KRAS, CDKN2A, ERBB2 or lineage-specific drivers	Mutation- and lineage-derived antigens	Variable PD-L1/B7-H3/TIM-3; inflamed, excluded or desert states possible	Do not pool distinct adenocarcinoma lineages (37, 38)
Anal SCC, HPV-associated	Persistent HPV16; immune compromise increases risk	E6/E7 plus PIK3CA and host changes	Viral plus host antigens	CD8-rich and chronic inflammatory-resistant phenotypes both occur	HPV prevalence in trials is not predictive evidence (44)
Anal SCC, HPV-independent	Rare nonviral pathway	Disruptive TP53 and other host alterations	Mutation-derived antigens	Poor CRT response may reflect apoptosis, hypoxia or exclusion	Requires prospective tissue collection (41, 43)
Vulvar SCC, HPV-associated	HPV-associated HSIL	p16 block; PI3K pathway; usually p53 wild-type pattern	E6/E7 plus host antigens	Not uniformly immune-hot	Separate from HPV-independent groups in trials
Vulvar SCC, HPV-independent	Lichen sclerosus, differentiated VIN, chronic injury and aging	TP53/CDKN2A/TERT/NOTCH or heterogeneous p53-wild-type programs	Mutation-, stress- and differentiation-derived antigens	Can contain PD-1+ T-cell/PD-L1+ macrophage niches or exclusion	Spatial niches are prognostic hypotheses, not predictive classes (48)
Vaginal SCC	HPV-associated majority; prior lower-genital neoplasia common	Poorly characterized	Viral and/or host antigens	Dedicated single-cell/spatial evidence is limited	Cross-site extrapolation must be explicit
Penile SCC, HPV-associated	Basaloid/warty pathway	E6/E7; basal/stem-like and LAMC2 programs	E6/E7 plus host antigens	May have reduced T cells and impaired DC costimulation	HPV does not guarantee presentation (52, 53)
Penile SCC, HPV-independent	Phimosis, lichen sclerosus, chronic inflammation	TP53, CDKN2A, NOTCH and differentiation programs	Host-derived antigens	May be infiltrated; specificity and function uncertain	Use HLA, TMB/MSI and spatial context rather than HPV alone

Evidence categories and priority labels are defined in the main text; author-proposed research tiers are not clinical guidelines.

## 7 Comparative cellular, stromal and metabolic immune ecosystems

The clinically relevant phenotype emerges from the full cancer-immunity cycle rather than from antigen source alone. A tumor may be antigen-rich but presentation-defective, immune-infiltrated but functionally suppressed, or genomically antigen-poor yet inflamed by non-tumor-specific responses. The conceptual model in **Figure 2** therefore separates spatial architecture from a functional suppressive overlay.

**Figure 2:**
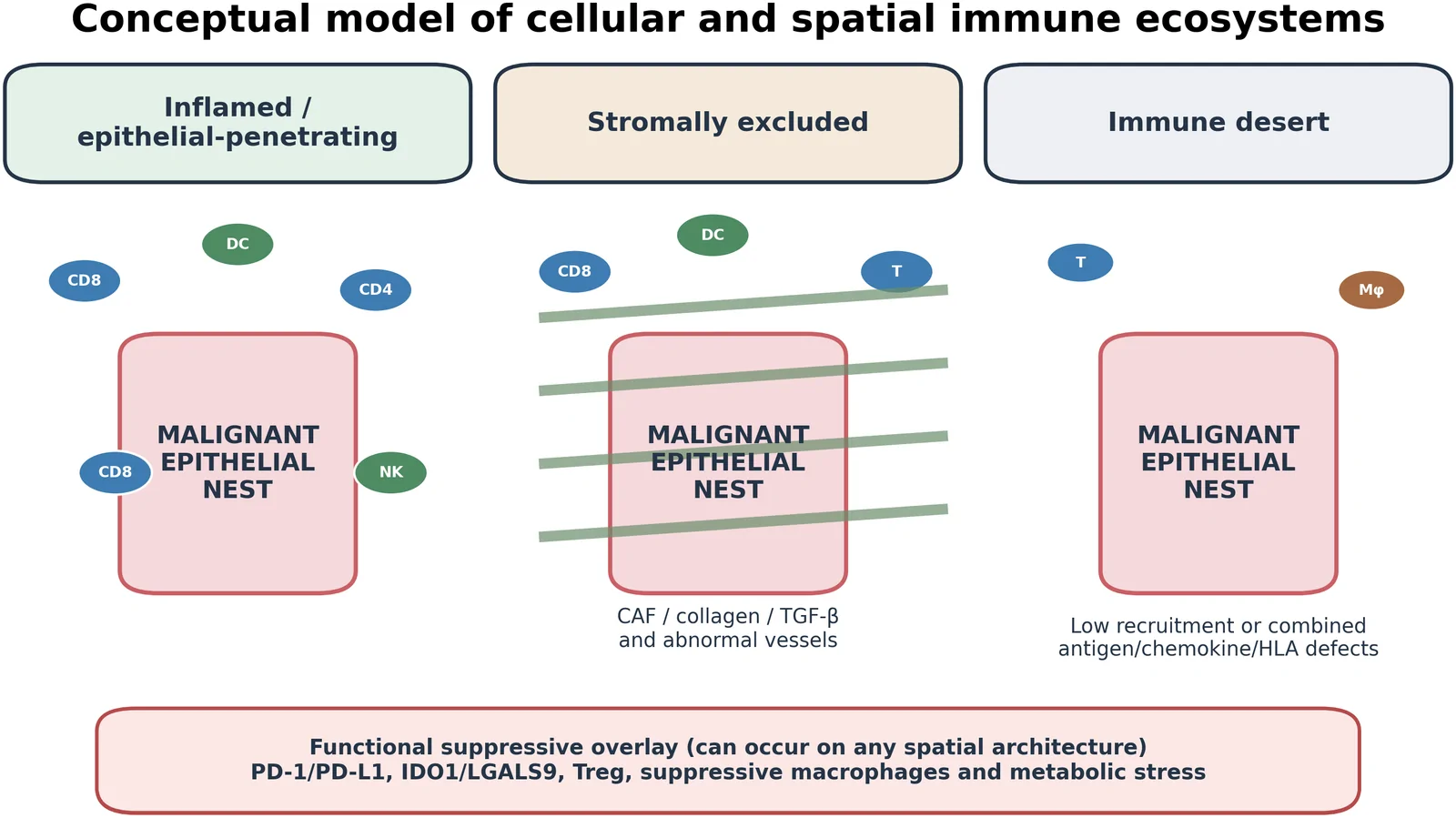
Conceptual model of cellular and spatial immune ecosystems. The image separates three spatial architectures—inflamed/epithelial-penetrating, stromally excluded and immune desert—from a functional suppressive overlay that may occur in any architecture. The diagram is a conceptual synthesis; evidence supporting individual elements and its limitations are described in the main text and **Table 3**. The categories are hypothesis-generating and are not validated clinical classes.

### 7.1 Antigen source and antigen-presentation competence

E6 and E7 have theoretical advantages as foreign, tumor-selective and functionally required antigens. Those advantages depend on active viral transcription, natural peptide processing, compatible HLA alleles, β2-microglobulin, transporter and proteasome function, and a T-cell repertoire capable of recognition. HPV DNA or p16 establishes none of these steps. HPV-independent tumors rely on mutation-, splice-, differentiation- or stress-derived antigens, which may be private and subclonal but can still support immunity when clonally expressed and efficiently presented.

HLA loss can be early, heterogeneous or treatment selected. Historical cervical and penile studies document classical or non-classical HLA alterations, and acquired resistance to checkpoint blockade in other cancers demonstrates that JAK/interferon pathway or antigen-presentation defects can emerge under therapy (17, 18, 57, 58). These general resistance mechanisms are biologically relevant but require direct confirmation in lower anogenital cohorts.

### 7.2 T-cell state, specificity and location

CD8 immunohistochemistry alone cannot distinguish tumor-reactive lymphocytes from bystander, tissue-resident, HPV-specific but non-tumor-directed, progenitor-exhausted or terminally dysfunctional cells. At least four dimensions are needed: density, state, specificity or clonality, and location. Progenitor-like exhausted states can be associated with checkpoint responsiveness in other tumors (59), but corresponding markers and thresholds have not been prospectively validated across lower anogenital sites.

Epithelial penetration is especially important. Lymphocytes confined to peritumoral stroma are not equivalent to cytotoxic cells in direct contact with malignant epithelium. General solid-tumor studies demonstrate how extracellular-matrix organization and TGF-β-associated fibroblast programs can restrict T-cell entry (60, 61). In lower anogenital cancers, direct evidence is strongest for descriptive spatial patterns, not for a validated treatment-selection threshold.

### 7.3 Dendritic and myeloid cells

Dendritic cells link antigen availability to adaptive immunity. HPV can reduce recruitment, maturation and costimulation, while tumor-derived cytokines can generate tolerogenic states. LAMP3-positive migratory dendritic cells may organize T-cell interactions but can also express PD-L1, IDO1 and regulatory chemokines. Cell abundance therefore does not establish effective cross-presentation. This distinction is critical for therapeutic vaccination, because circulating E6/E7-specific T-cell expansion cannot compensate for failed local priming or absent peptide–HLA display.

Macrophages and monocytes occupy inflammatory, antigen-presenting, lipid-metabolic and matrix-remodeling states that cannot be represented adequately by the M1/M2 dichotomy. SPP1-, APOC1- and C1QC-associated labels recur across studies, but similar gene labels do not prove identical cell origin or function when platforms, tissue handling and disease sites differ. Direct perturbation is uncommon. Myeloid orchestration of systemic and local suppression has been demonstrated in HPV-positive experimental models (62), while human vulvar and cervical studies chiefly provide spatial or transcriptional associations.

### 7.4 B cells and tertiary lymphoid organization

B-lineage cells may support antigen presentation, antibody production and local T-cell priming, whereas regulatory B-cell states can suppress immunity. Dispersed plasma cells, naive B-cell clusters and mature tertiary lymphoid structures are not interchangeable. Cervical and penile cohorts suggest prognostic associations (34, 55), but structure definitions, tissue regions and scoring methods differ. Any proposed predictive role should be evaluated prospectively and should demonstrate incremental value beyond stage, histology and PD-L1.

### 7.5 Fibroblasts, extracellular matrix and vascular access

Cancer-associated fibroblasts regulate immunity through collagen and laminin deposition, tissue stiffness, TGF-β, CXCL12, inflammatory cytokines and vascular remodeling. Fibroblast states are heterogeneous; some may restrain invasion while others create exclusionary tracks. Cervical PLOD2-associated programs and penile LAMC2–integrin signaling illustrate matrix-linked malignant states (27, 53), but most evidence remains transcriptomic or computational. Broad fibroblast depletion is therefore not justified by these observations.

Abnormal vessels, high interstitial pressure and hypoxia further restrict immune-cell entry. Antiangiogenic therapy can improve perfusion in selected contexts, but vascular normalization is dynamic and dose dependent. Tissue accessibility at vulvar, vaginal, penile and anal sites may facilitate local intervention and repeated biopsy, although this logistical advantage has not yet translated into validated spatially guided regimens.

### 7.6 Immunometabolism

Hypoxia, lactate, adenosine, nutrient competition and lipid handling can suppress immune function. Lactate accumulation can impair T- and NK-cell activity, glucose competition can limit effector metabolism, and adenosine signaling can inhibit multiple immune compartments (31–33). Cervical spatial studies identify metabolic regions and candidate lactate-associated programs (29, 30), but direct causal evidence in lower anogenital tumors remains limited. Metabolic markers should therefore be treated as exploratory biomarkers or combination hypotheses, not as established predictors.

### 7.7 Spatial immune architectures and functional overlay

Three spatial architectures—an inflamed/epithelial-penetrating pattern, a stromally excluded pattern, and an immune-desert pattern—are supported by established tumor-immunity frameworks and direct lower-anogenital observations (22, 24, 47, 53, 60, 61, 102, 103). The previously proposed category “inflamed but suppressed” is better treated as a functional overlay rather than a separate spatial architecture. Checkpoint expression, regulatory T cells, suppressive macrophages, IDO1/LGALS9 and metabolic stress can occur in an epithelial-penetrating or excluded tumor and, less commonly, within sparse immune infiltrates.

An inflamed/epithelial-penetrating tumor contains lymphocytes within malignant nests and is the architecture most compatible with pre-existing immunity, but response still depends on antigen specificity and reversible dysfunction (22, 24, 47, 53, 102, 103). A stromally excluded tumor accumulates immune cells at invasive margins or fibroblast-rich regions without epithelial access (47, 53, 60, 61, 102, 103). An immune desert shows limited recruitment because of low antigenicity, chemokine silencing, HLA loss, vascular dysfunction or combined defects (102, 103). These are conceptual research categories, not validated clinical classes. Their prevalence, thresholds, reproducibility and predictive utility must be tested separately by site and treatment setting.

## 8 Methodological appraisal of single-cell and spatial evidence

Single-cell and spatial technologies are central to this Review but are also a major source of overinterpretation. **Table 3** summarizes representative studies, sample numbers, platforms, validation and limitations. Findings are classified as transcriptional association, computational communication inference, spatial colocalization, protein-level validation, functional perturbation or clinical association.

**Table 3:** Methodological appraisal of representative single-cell and spatial studies.

Cancer/site (reference)	Patients/specimens and treatment state	Platform/material	HPV ascertainment	Main reported finding	Evidence level	Principal limitations
Cervical SCC (22)	5 samples for snRNA-seq; 18 spatial samples; treatment context varied	snRNA-seq on frozen tissue; Stereo-seq	Study-specific HPV/pathology annotation	LGALS9 and IDO1 spatial programs	Transcriptional association and spatial localization	Different specimen sets; nuclear transcript bias; cross-sample integration
Cervical progression (23)	9 scRNA-seq specimens: 2 uninfected normal, 2 HPV-positive normal, 2 HPV-positive HSIL and 3 HPV-positive cervical cancers; plus 2 cervical-cancer spatial specimens. Dataset overlap was not clearly specified.	scRNA-seq (GSE208653) plus 10x spatial transcriptomics (GSE208654)	Dataset labels: NO_HPV, N_HPV, HSIL_HPV and CA_HPV	SPP1+ macrophage expansion; inferred SPP1-CD44 communication	Computational communication inference; spatial association	Small public-data cohort; only 2 spatial cancers; dataset overlap unclear; no receptor perturbation; stage/batch confounding
HPV-positive versus HPV-negative cervix (24)	7 untreated biopsies; scRNA component included 2 HPV+ and 2 HPV− tumors	10x scRNA-seq and Visium; fresh biopsy	HPV DNA genotyping plus p16	Distinct cDC2, T-cell and epithelial-myeloid programs; inferred ANXA1-FPR and MDK-LRP1	Small-cohort transcriptional and computational association	Patient n very small; spots contain many cells; no functional perturbation
Cervical histology comparison (26)	8 tumors: 5 SCC and 3 adenocarcinomas	snRNA-seq	HPV-related cervical cancers	Histology-specific malignant and immune states	Transcriptional association	Lineage and patient effects; no spatial validation
Vulvar SCC (47)	4 early-stage cases spanning HPV-dependent and independent disease	Spatial transcriptomics	Study-specific HPV/p16 classification	Different epithelial, collagen and inflammatory programs	Spatial association	Extremely small cohort; spot admixture; no single-cell reference or perturbation
Penile SCC (53)	6 patients; 6 snRNA-seq samples and 4 spatial samples; untreated primary tumors	snRNA-seq and Stereo-seq on frozen tissue	2 HPV-associated and 4 HPV-independent patients	LAMC2-high boundary state; inferred laminin-integrin signaling	Transcriptional/spatial association	Small unbalanced groups; inferred interaction; no intervention

Evidence categories and priority labels are defined in the main text; author-proposed research tiers are not clinical guidelines.

Fresh-tissue dissociation can induce immediate-early and stress-response genes and preferentially lose fragile granulocytes, adipocytes or stromal cells (63, 64). Single-nucleus RNA sequencing enables frozen-tissue analysis and may preserve difficult cell populations, but it captures nuclear rather than whole-cell transcripts and can underrepresent cytoplasmic activation markers. Direct comparisons show that single-cell and single-nucleus workflows have cell-type-specific biases (65). Therefore, apparent differences between studies may reflect preparation rather than biology.

Patient number, not cell number, determines biological replication. Differential expression based on thousands of cells from two or three patients can generate artificially small P values if donor effects are ignored. Pseudobulk or mixed-effects analyses are preferable, and pseudoreplication should be explicitly reported (66). Batch correction can remove technical variation but may also erase genuine site or treatment differences. Cell-state annotation depends on selected markers and reference atlases; copy-number inference identifies probable malignant cells but is less reliable in low-aneuploid or normal-like tumors.

Ligand–receptor tools infer that a ligand and receptor are expressed in candidate sender and receiver populations. They do not demonstrate secretion, protein binding, receptor activation, directionality or biological necessity. Spatial colocalization strengthens plausibility but remains associative. Orthogonal protein imaging, receptor phosphorylation, ex vivo perturbation, genetic or pharmacological interruption and clinical replication represent progressively stronger evidence. Accordingly, SPP1–CD44, MDK–LRP1 and LAMC2–integrin programs are described here as inferred or associated unless direct perturbation was performed.

Spatial transcriptomic platforms vary from multicellular spots to near-cellular or subcellular resolution. Spot admixture can create apparent co-expression between cells that never interact, and deconvolution depends on the accuracy of the reference single-cell dataset (67). Small tissue regions may miss invasive margins, tertiary lymphoid structures or hypoxic cores. Treatment exposure, tissue fixation and primary versus metastatic origin must be recorded. Similar macrophage or fibroblast labels across studies should be considered candidate conserved programs only after harmonized analysis and orthogonal validation.

## 9 Clinical translation and evidence hierarchy

Clinical evidence is separated from mechanistic interpretation in **Figure 3** and **Table 4**. “Established” refers only to randomized evidence and/or a named regulatory or guideline context. A predominantly HPV-associated trial population does not establish HPV as a predictive biomarker, and US regulatory decisions are not universal treatment standards.

**Figure 3:**
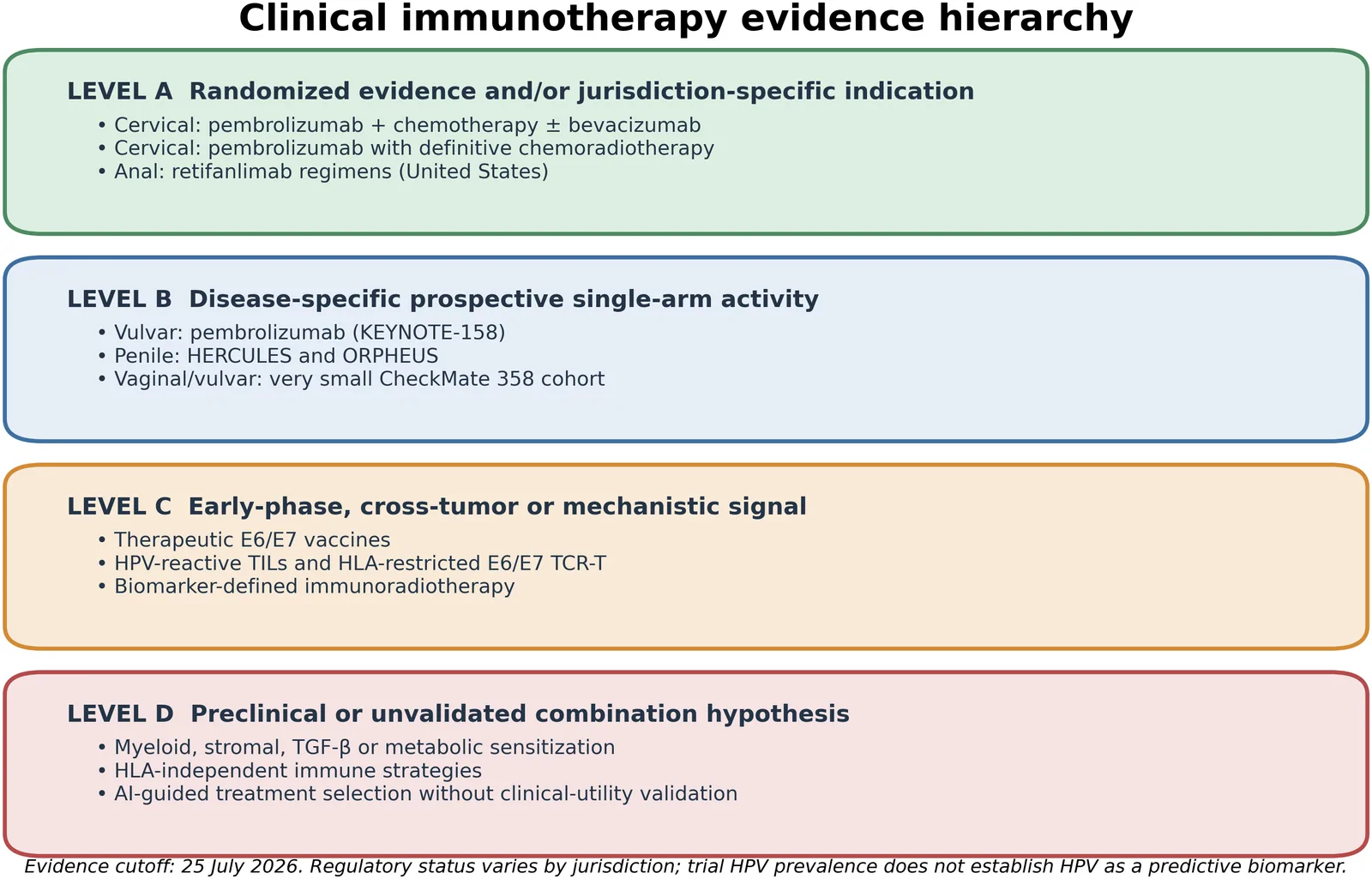
Clinical immunotherapy evidence hierarchy. Level A includes randomized evidence and/or a named jurisdiction-specific indication; Level B, disease-specific prospective single-arm activity; Level C, early-phase, cross-tumor or mechanistic signals; and Level D, preclinical or unvalidated combination hypotheses. HPV prevalence within a trial population does not establish HPV status as a predictive biomarker. Evidence cutoff: 25 July 2026.

**Table 4:** Representative immune-checkpoint trials and evidence classification (cutoff 25 July 2026).

Cancer/trial	Setting/design	Regimen	N	Key result	Evidence category	Jurisdiction/interpretation
Cervical KEYNOTE-826	First-line persistent/recurrent/metastatic; phase III	Pembrolizumab + chemotherapy ± bevacizumab	617	CPS ≥1 OS improvement	Randomized predictive/regulatory evidence	Practice-changing in eligible PD-L1-positive disease (68, 69)
Cervical KEYNOTE-A18	High-risk locally advanced; phase III	Pembrolizumab + CRT then maintenance	1,060	PFS and OS benefit	Randomized evidence	US approval initially FIGO 2014 stage III–IVA; status varies by jurisdiction (70, 71)
Cervical CALLA	Locally advanced; phase III	Durvalumab +/after CRT	770	Primary PFS endpoint not met	Negative randomized evidence	Do not generalize immuno-CRT combinations (72)
Cervical EMPOWER-Cervical 1	After platinum; phase III	Cemiplimab vs chemotherapy	608	OS 12.0 vs 8.5 months	Positive randomized evidence	Availability varies by jurisdiction (73)
Anal POD1UM-303	Untreated inoperable recurrent/metastatic; phase III	Retifanlimab + carboplatin/paclitaxel	308	PFS and ORR improvement	Randomized evidence	US first-line approval in 2025 (78, 79)
Anal NCI9673 Part B	Refractory; randomized phase II	Nivolumab ± ipilimumab	100	No significant efficacy improvement; more toxicity	Negative randomized phase II	Does not support unselected dual-checkpoint escalation (80)
Vulvar KEYNOTE-158	Previously treated advanced; phase II	Pembrolizumab	101	ORR 10.9%; some durable responses	Prospective single-arm activity	Signal in a minority; no validated HPV/p53 predictor (81)
Vaginal/vulvar CheckMate 358	Recurrent/metastatic; phase I/II	Nivolumab	5	One response	Very small signal-generating cohort	No site-specific standard (82)
Penile HERCULES	First-line advanced; single-arm phase II	Pembrolizumab + platinum chemotherapy	37	ORR 39.4%	Prospective single-arm activity	Requires randomized confirmation (83)
Penile ORPHEUS/PERICLES	Advanced; phase II studies	Retifanlimab or atezolizumab ± RT	18/32	Modest activity; no routine standard	Prospective exploratory activity	No established jurisdiction-wide checkpoint standard (84, 85)

Evidence categories and priority labels are defined in the main text; author-proposed research tiers are not clinical guidelines.

### 9.1 Cervical cancer

In persistent, recurrent or metastatic cervical cancer, pembrolizumab added to platinum-based chemotherapy with or without bevacizumab improved progression-free and overall survival in KEYNOTE-826, establishing a first-line option for eligible PD-L1-positive disease in multiple jurisdictions (68, 69). Chemotherapy-associated antigen release and bevacizumab-associated vascular effects are plausible contributors, but the randomized trial did not prove these mechanisms.

KEYNOTE-A18 demonstrated progression-free and overall-survival benefit when pembrolizumab was combined with definitive chemoradiotherapy and continued as maintenance for high-risk locally advanced disease (70). The US Food and Drug Administration initially approved the regimen for FIGO 2014 stage III–IVA disease in January 2024 (71). In contrast, CALLA did not meet its primary endpoint when durvalumab was added to chemoradiotherapy in an unselected population (72). These divergent trials show that checkpoint target, population, treatment timing and maintenance cannot be treated as interchangeable.

Cemiplimab improved survival over investigator-choice chemotherapy after platinum therapy (73), and pembrolizumab monotherapy produced durable responses in a minority of previously treated PD-L1-positive tumors (74). PD-L1 is therefore a clinically useful enrichment marker in defined settings but not a complete predictor.

### 9.2 Anal, vulvar, vaginal and penile cancers

Nivolumab and pembrolizumab established prospective single-arm checkpoint activity in previously treated metastatic anal carcinoma (75, 76). Retifanlimab monotherapy produced modest activity after platinum therapy (77), while POD1UM-303/InterAACT-2 provided randomized evidence that retifanlimab plus carboplatin–paclitaxel improved progression-free survival and response rate in previously untreated inoperable recurrent or metastatic disease (78). The combination and a later-line monotherapy indication were approved in the United States in 2025 (79). HPV positivity was common in these studies, but that prevalence does not prove HPV status predicts benefit. Adding ipilimumab to nivolumab did not significantly improve efficacy and increased toxicity in NCI9673 Part B (80).

In vulvar cancer, KEYNOTE-158 reported an objective response rate near 11%, with some durable responses (81). CheckMate 358 included only five vaginal or vulvar patients, making the one observed response an imprecise signal rather than a site-specific standard (82). In penile cancer, HERCULES reported encouraging single-arm chemoimmunotherapy activity, whereas ORPHEUS and PERICLES showed modest checkpoint activity and did not establish routine standards (83–85). Future trials should prespecify HPV/p16, p53, histology, HLA integrity and spatial immune variables rather than relying on *post hoc* subgroup interpretation.

### 9.3 Therapeutic HPV vaccines

Preventive HPV vaccines do not treat established E6/E7-expressing cancer. Therapeutic platforms include synthetic long peptides, DNA or RNA constructs, bacterial or viral vectors and dendritic-cell products. In cervical intraepithelial neoplasia, VGX-3100 demonstrated that E6/E7-directed vaccination can induce regression in a randomized setting (86). In invasive disease, vaccine monotherapy is frequently limited by antigen-presentation loss, weak local priming, exhaustion and exclusion.

ISA101 plus nivolumab produced responses in an HPV16-positive cross-tumor phase II cohort dominated by head-and-neck cancer (87), and GX-188E plus pembrolizumab showed activity in HPV16/18-positive advanced cervical cancer (88). Axalimogene filolisbac demonstrated feasibility but did not establish a standard (89). These studies support biological activity, not a universal vaccine strategy. Trials should document E6/E7 transcription, HLA compatibility, intratumoral trafficking and expansion of target-specific clones rather than peripheral immunogenicity alone.

### 9.4 Tumor-infiltrating lymphocytes and TCR-engineered therapy

HPV-reactive tumor-infiltrating lymphocyte therapy produced durable complete regressions in selected metastatic cervical cancers (90). Activity may derive from viral and host neoantigen recognition; product clonality, differentiation and cytotoxicity are as important as the measured frequency of HPV-reactive cells. Manufacturing, lymphodepletion and tissue availability remain major limitations for rare cancers.

E6- and E7-directed T-cell receptor-engineered therapies provide an HLA-restricted route to intracellular viral antigens. First-in-human E6 and E7 studies produced objective responses but identified HLA loss and interferon-pathway defects as resistance mechanisms (91, 92). The 2026 KITE-439 phase I trial reported only one transient partial response among eight patients and could not confirm target peptide presentation in tested tumors; one tumor had lost both relevant HLA-A*02:01 alleles (93). Candidate selection therefore requires genotype, viral transcription, compatible HLA, HLA/β2-microglobulin integrity and, when feasible, direct peptide–HLA evidence. HPV DNA alone is insufficient.

### 9.5 Combination strategies and HPV-independent sensitization

Radiotherapy and cytotoxic therapy can release antigens and modify presentation, but they can also deplete lymphocytes, induce TGF-β and recruit suppressive myeloid cells. The KEYNOTE-A18/CALLA contrast demonstrates that a general “radiation plus immunotherapy” label is biologically inadequate. Sequence, dose distribution, lymphocyte exposure and maintenance matter.

No sensitization strategy has been validated specifically for HPV-independent lower anogenital tumors. Epigenetic or innate agonists may restore reversible presentation defects, whereas biallelic B2M or HLA loss is unlikely to be rescued by interferon. DNA-damage combinations are plausible in replication-stressed tumors, but pembrolizumab plus olaparib had limited objective activity in GOTIC-025 (94). Myeloid, TGF-β, vascular, matrix or metabolic combinations remain preclinical or early phase and should be matched to a demonstrated bottleneck rather than applied empirically.

## 10 Tiered biomarker framework

The previous “minimum practical panel” was neither minimal nor routinely practical. **Table 5** and **Figure 4** reorganize the proposal into three tiers. Tier 1 contains current clinical core assays available in routine pathology or validated molecular laboratories. Tier 2 contains extended translational tests that may support trials or selected complex decisions. Tier 3 contains exploratory single-cell, spatial, peptide–HLA and artificial-intelligence methods. Priority labels are author-proposed and are not clinical recommendations.

**Table 5:** Three-tier biomarker framework for clinical, translational and exploratory use.

Tier/assay	Intended use	Role	Specimen	Analytical maturity	Setting/evidence	Unresolved issues
Tier 1: site/histology + HPV/p16/p53	Etiological and lineage classification	Diagnostic/prognostic	FFPE tissue	Routine if site-appropriate	Clinical pathology core	Discordance, false negatives and central review
Tier 1: PD-L1 CPS/TPS	Eligibility in defined regimens	Predictive only in specified settings	FFPE tissue	Validated assays available	Defined cervical pembrolizumab regimens, including CPS ≥1 persistent/recurrent/metastatic disease	Cutoff, assay and cellular source
Tier 1: MMR/MSI and standard drivers	Identify tissue-agnostic sensitivity or targetable subtype	Predictive	FFPE tissue/blood depending assay	Routine molecular pathology	Especially relevant in HPV-independent disease	Low prevalence; panel/site differences
Tier 2: E6/E7 RNA	Confirm transcriptionally active HPV	Diagnostic/enrichment	RNA-preserved FFPE or fresh tissue	Specialized but established	Vaccine/TCR trial eligibility and discordant cases	RNA quality and genotype coverage
Tier 2: tumor genomics + HLA-I/B2M/APM	Assess host drivers and presentation competence	Predictive hypothesis; essential for TCR-T	FFPE/fresh tissue and germline HLA sample	Specialized	Cellular therapy and resistance studies	Allele-specific loss and functional presentation
Tier 2: multiplex immune profiling	Resolve T-cell/myeloid state and penetration	Prognostic/translational	Well-oriented FFPE tissue	Moderate; platform dependent	Prospective correlative studies	Marker panels and thresholds not standardized
Tier 2: ctHPV DNA or tumor-informed ctDNA	Residual disease and response monitoring	Prognostic/monitoring	Serial plasma	Emerging validation	Post-CRT cervical/anal studies	Assay harmonization and intervention thresholds (95–97)
Tier 3: single-cell/spatial profiling	Discover bottlenecks and pharmacodynamics	Exploratory	Fresh/frozen/optimized FFPE	Research	Mechanism-focused trials	Cost, bias, sampling, pseudoreplication
Tier 3: peptide-HLA/TCR assays	Verify target display and recognition	Exploratory predictive	Fresh tumor/blood	Research-intensive	TCR-T and vaccine programs	Sensitivity, HLA restriction, tumor heterogeneity
Tier 3: AI multimodal models	Prescreen or integrate morphology and biomarkers	Exploratory decision support	Digitized slides plus clinical/molecular data	Early research	No validated treatment-selection role	Leakage, domain shift, calibration, fairness, external validation

Evidence categories and priority labels are defined in the main text; author-proposed research tiers are not clinical guidelines.

**Figure 4:**
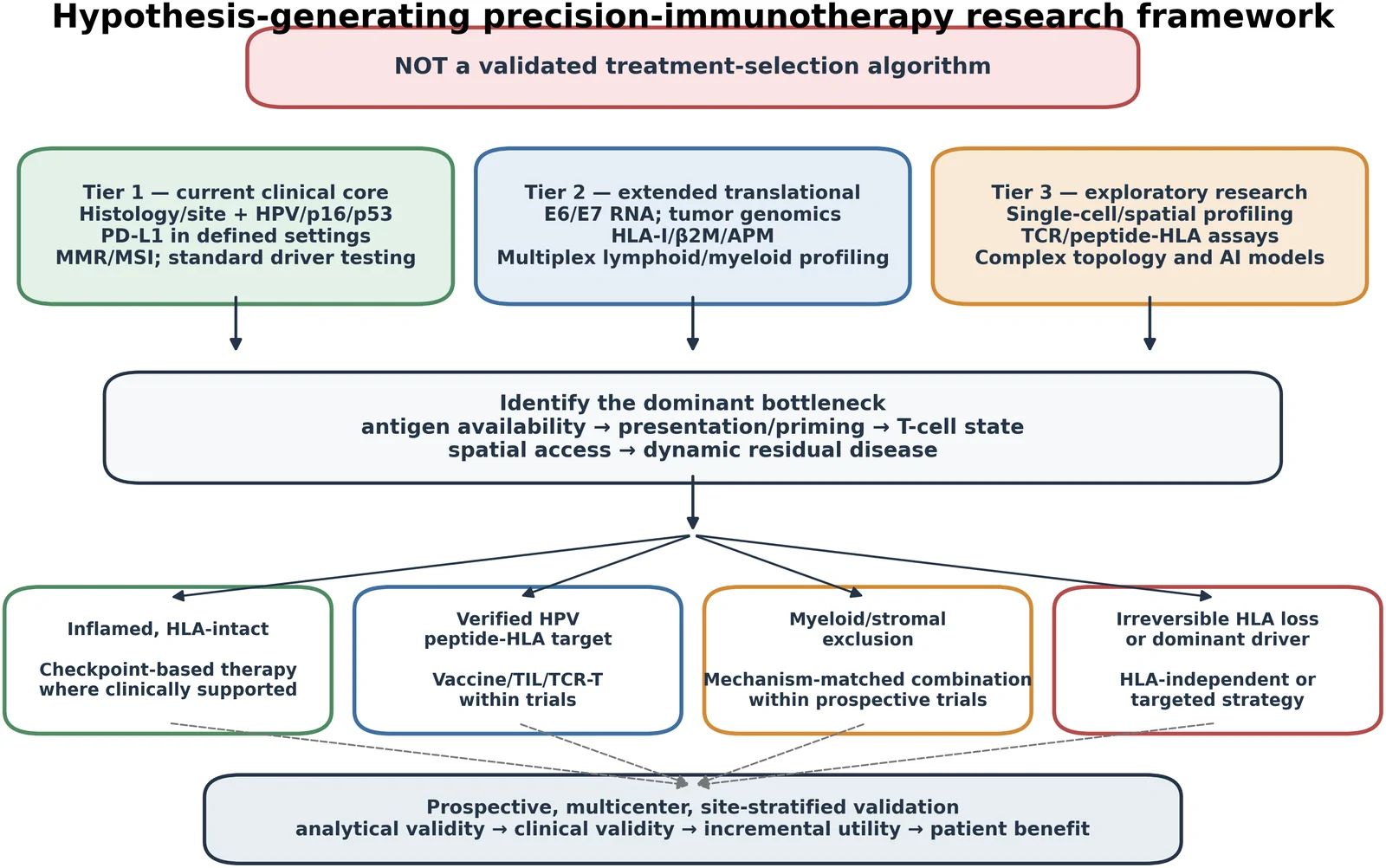
Hypothesis-generating precision-immunotherapy research framework. The three tiers separate current clinical core assays from extended translational profiling and exploratory single-cell, spatial, peptide–HLA and artificial-intelligence methods. The framework aims to identify the dominant bottleneck and nominate a research strategy. It is not a validated treatment-selection algorithm. Every branch requires prospective, multicenter and site-stratified validation of analytical validity, clinical validity, incremental utility and patient benefit.

Tier 1 begins with site and histology, site-appropriate HPV/p16/p53 classification, PD-L1 in settings where it determines eligibility, mismatch-repair or microsatellite-instability testing, and standard driver testing for relevant HPV-independent histologies. The intended use, specimen, treatment setting and jurisdiction should be stated for every assay. PD-L1 is predictive only in defined treatment contexts; HPV and p16 are primarily etiological or prognostic rather than universally predictive.

Tier 2 includes E6/E7 RNA, broader tumor genomics, HLA-I/β2-microglobulin and antigen-processing assessment, multiplex lymphoid/myeloid phenotyping and validated circulating tumor DNA assays. HLA testing is essential for HLA-restricted cellular therapy but not yet a routine checkpoint biomarker. Circulating tumor HPV DNA is emerging as a prognostic and monitoring tool after chemoradiotherapy in cervical and anal cancer (95–97); intervention thresholds and assay harmonization remain unresolved.

Tier 3 includes single-cell and spatial profiling, T-cell receptor or peptide–HLA assays, complex topology measures and artificial-intelligence integration. These methods can identify research hypotheses and pharmacodynamic changes, but prospective analytical validity, clinical validity, incremental utility and patient benefit must be demonstrated before they can guide treatment.

## 11 Digital pathology and artificial intelligence

Artificial intelligence is retained as an emerging, bounded component rather than a central validated pillar. Relevant tasks include prediction of etiological or molecular subtype, quantification of immune-cell distribution, identification of stromal exclusion and multimodal response modeling. A 2025 cervical study used histopathology to predict consensus molecular subtypes across three cohorts, illustrating feasibility but not immunotherapy-selection utility (98). No current model has established that routine hematoxylin–eosin morphology can replace direct measurement of lower anogenital immune topology.

Ground-truth labels require particular scrutiny. HPV DNA, p16, E6/E7 RNA, molecular subtype and treatment response are not interchangeable outcomes. Patient-level and institution-level separation is required to prevent leakage; scanner, stain, fixation and specimen-processing differences create domain shift. Class imbalance is severe for vaginal cancer and rare HPV-independent lineages. Internal cross-validation is not external validation. Models should report discrimination, calibration, uncertainty, subgroup performance and decision-curve analysis and should be compared with existing clinical standards rather than with chance alone.

TRIPOD+AI provides reporting guidance for prediction models, PROBAST+AI addresses risk of bias and applicability, and CONSORT-AI applies to prospective interventional trials (99–101). External validation across institutions, populations and anatomical sites is mandatory. Explainability maps can identify regions associated with a prediction but do not prove that the highlighted morphology is causal. AI should be used initially for prescreening or research enrichment, not as an autonomous treatment-selection system.

## 12 Future trial design

Future studies should begin with validated etiological and histological classification, then identify the dominant bottleneck. An HLA-intact, inflamed tumor in a clinically supported setting may be suitable for checkpoint blockade. A transcriptionally HPV-driven but weakly primed tumor may be considered for vaccination within a trial. A checkpoint-refractory tumor with verified peptide–HLA display may be eligible for TCR-engineered therapy. Irreversible HLA loss requires HLA-independent or oncogene-directed strategies. These branches are research hypotheses, not a validated algorithm.

Rare-site trials require international collaboration, centralized pathology and prospective tissue collection. Basket enrollment by HPV association can improve recruitment, but site and lineage must remain stratification variables. Randomized phase II designs with a prespecified mechanism-of-action endpoint are preferable to large biomarker-light single-arm studies. Longitudinal tissue, blood and circulating tumor DNA should test whether the intended bottleneck was actually modified.

Validation should proceed through analytical validity, clinical validity, incremental utility and patient benefit. A biomarker workflow should not be adopted merely because it predicts an existing marker or correlates with outcome retrospectively. It must improve decision making beyond pathology, stage and validated molecular standards, and its benefit should be shown in multicenter, site-stratified prospective studies.

## 13 Discussion and limitations

The principal contribution of this Review is to separate etiological origin from functional immune phenotype. HPV association supplies a shared viral-antigen source but does not guarantee transcription, presentation, priming or spatial access. HPV-independent tumors lack constitutive viral antigens but are not necessarily immune silent. This distinction is supported directly by cervical, vulvar and penile observations, although the density and quality of evidence vary markedly by site.

The proposed spatial framework deliberately revises the earlier four-architecture model. Inflamed/epithelial-penetrating, stromally excluded and immune-desert states describe spatial organization. “Inflamed but suppressed” is better understood as a functional overlay that can coexist with an inflamed or excluded architecture. This formulation aligns more closely with established solid-tumor models and prevents checkpoint expression or macrophage abundance from being mistaken for a separate topology. The categories remain conceptual; no validated cutoff defines epithelial penetration, exclusion or desert status in these diseases.

Several limitations constrain the synthesis. First, cervical cancer dominates the literature and includes more randomized trials and multi-omic datasets than vaginal, vulvar or penile cancer. Cross-site extrapolation was therefore minimized but cannot be eliminated. Second, histological and anatomical heterogeneity is substantial, especially when HPV-independent cervical adenocarcinomas are compared with squamous tumors. Third, many cohorts are retrospective, geographically concentrated and small; immune-compromised populations are incompletely represented despite their high disease burden.

Fourth, HPV ascertainment differs across studies. HPV DNA, PCR, DNA *in situ* hybridization, E6/E7 RNA, p16 and morphology have different analytical meanings, and false-negative mechanisms are not uniformly investigated. Fifth, single-cell and spatial studies frequently include few patients, use different preparation platforms, and are vulnerable to dissociation bias, pseudoreplication, batch effects and spot admixture. Recurrent labels such as SPP1-positive macrophage or LAMC2-high tumor cell may indicate conserved programs, but they may also reflect annotation conventions or technical convergence.

Sixth, treatment-related remodeling complicates comparison of untreated primary tissue with post-radiotherapy, recurrent or metastatic specimens. Seventh, the clinical evidence hierarchy is jurisdiction dependent and evolves rapidly; regulatory status was reviewed through 25 July 2026. Finally, the tiered biomarker workflow and AI component are hypothesis-generating. Assay combinations, cutoffs, turnaround time, cost, accessibility and incremental utility are undefined. The framework should not be interpreted as a clinical treatment-selection algorithm.

These limitations define a practical research agenda. The decisive next step is not to add every available assay to every patient, but to prospectively test whether one clearly defined bottleneck and its matched measurement improve treatment selection within a specific site and disease setting.

## 14 Conclusions

HPV-associated and HPV-independent lower anogenital tract malignancies begin with different oncogenic and antigenic programs but can converge on the same immune-evasion bottlenecks. HPV association is an evolutionary and etiological descriptor, not a fixed immune phenotype. HPV independence does not imply an immune desert.

Immune checkpoint inhibitors have established roles in defined cervical and anal cancer settings, whereas evidence in vulvar, vaginal and penile cancer is more limited. Therapeutic vaccines, tumor-infiltrating lymphocytes, T-cell receptor-engineered therapies and myeloid, stromal or metabolic sensitization remain investigational or context dependent. Computational ligand–receptor inference and spatial colocalization should not be written as functional causality.

The proposed three-tier biomarker framework and spatial architectures are hypothesis-generating research tools. They are not validated clinical classes, and assay combinations and thresholds remain undefined. Prospective, multicenter, site- and lineage-stratified studies must demonstrate analytical validity, clinical validity and incremental utility beyond existing pathological and molecular standards before the framework can influence routine treatment selection.
